# Small RNA sequencing reveals microRNAs related to neuropathic pain in rats

**DOI:** 10.1590/1414-431X20198380

**Published:** 2019-09-16

**Authors:** Dawei Dai, Junyu Wang, Ying Jiang, Lei Yuan, Youming Lu, Aijun Zhang, Dongdong Zou, Xin Chen

**Affiliations:** 1 Department of Neurosurgery, Changzheng Hospital, Naval Medical University, Neurosurgical Institute of Shanghai, Neurosurgical Institute of PLA, Shanghai, China; 2 Department of Neurosurgery, The Affiliated Sixth People's Hospital, Shanghai Jiaotong University, Shanghai, China

**Keywords:** MicroRNAs, Neuropathic pain, Spared nerve injury, Dorsal root ganglion

## Abstract

The present study aimed to identify microRNAs (miRNAs) that are involved in neuropathic pain and predict their corresponding roles in the pathogenesis and development process of neuropathic pain. The rat model of neuropathic pain caused by spared nerve injury (SNI) was established in Sprague-Dawley male rats, followed by small RNA sequencing of the L3–L6 dorsal root ganglion. Real-time PCR was performed to validate the differently expressed miRNAs. Functional verification was performed by intrathecally injecting the animals with miRNA agomir. A total of 72 differentially expressed miRNAs were identified in the SNI rats, including 33 upregulated and 39 downregulated miRNAs. The results of qPCR further verified the expression levels of rno-miR-6215 (P=0.015), rno-miR-1224 (P=0.030), rno-miR-1249 (P=0.038), and rno-miR-488-3p (P=0.048), which were all significantly downregulated in the SNI rats compared to the control ones. The majority of differentially expressed miRNAs were associated with phosphorylation, intracellular signal transduction, and cell death. Target prediction, Gene Ontology, and Kyoto Encyclopedia of Genes and Genomes pathway enrichment analyses suggested that these differentially expressed miRNAs targeted genes that are related to axon guidance, focal adhesion, and Ras and Wnt signaling pathways. Moreover, miR-1224 agomir significantly alleviated SNI-induced neuropathic pain. The current findings provide new insights into the role of miRNAs in the pathogenesis of neuropathic pain.

## Introduction

Neuropathic pain is caused by injury to the peripheral or central nervous system, and is typically chronic (1). Chronic pain stimulation induces epigenetic abnormalities in transcription, translation, and post-translational modifications in related cells depending on the degree of injury or the type of disease (2). Some studies have indicated that receptors, multiple neurotransmitter systems, ionic channels, and various cell types in the peripheral and/or central nervous system contribute to the pathogenesis of neuropathic pain (3). A meta-analysis found that approximately four to ten front-line neuropathic pain drugs are needed to achieve 50% pain relief in most positive trials (4). New therapeutic targets and more effective treatments for neuropathic pain are urgently needed (5). Therefore, there is a need to study the pathogenesis and mechanisms underlying the treatment of neuropathic pain, as well as to identify potential therapeutic targets or develop treatment strategies.

Recently, important progress in research on the molecular mechanisms underlying neuropathic pain has been made. Studies have shown that microRNAs (miRNAs) play important roles in regulating pain transmission in animal experiments and clinical studies. For example, miR-23b can regulate MOR1 expression, and the opioid resistance of patients could be associated with the upregulation of miR-23b expression (6). MiR-146a expression was reported to be upregulated in the spinal cord after spinal nerve ligation, and replenishing intrathecal mimic of miR-146a further reduced allodynia. Wang et al. showed that miR-146a-5p can relieve neuropathic pain by inhibiting the *IRAK1/TRAF6* signaling pathway (7). Willemen et al. (8) showed that miR-124 can reverse M1/M2 balance by exerting anti-inflammatory effects on the M2 phenotype while reducing mechanical hyperalgesia and pain behavior. The above findings suggest that miRNAs play important roles in neuropathic pain.

In the current study, we generated a rat model of spared nerve injury (SNI). The miRNA expression profiles of SNI rats were analyzed by small RNA sequencing, and important miRNAs involved in SNI rats were screened. Differentially expressed miRNA target genes were predicted based on bioinformatics analyses. Further, real-time PCR (qPCR) was performed to verify the differentially expressed miRNAs. The present study identified novel targets and could serve as the basis for the development of therapeutic strategies for subsequent neuropathic pain treatment.

## Material and Methods

### Animals

Sprague-Dawley (SD) male rats weighing 220–280 g were acquired from Shanghai Sippe-Bk Lab Animal Co., Ltd. (China). Animals were randomly assigned to the treatment or sham-operated group, with three rats in each group. The rats were maintained in a specific pathogen-free environment with 12-h light-dark cycles. Rats were fed rodent feed and water. These experiments were approved by Changzheng Hospital, Naval Medical University (China).

### Spared nerve injury model

The SD rats were allowed to adapt to the conditions for a week, following which the SNI model was generated as previously described in the literature (9). Briefly, the rats were anesthetized with 10% chloral hydrate (0.3 mL/100 g, Sigma-Aldrich Quimica, Spain), and were bundled in the prone position. The skin of the right hind limbs of each rat was cut, and the muscles were separated; the trunk of the sciatic nerve and its distal branch were fully exposed. The tibial nerve and the common peroneal nerve were ligated and clipped. The sural nerve was preserved and then sutured by layer. In the sham-operated group, the skin of the right hind limb was cut, the muscles were separated, and the skin was sutured immediately after exposing the sciatic nerve. After one week, all rats were euthanatized by intraperitoneally injecting with pentobarbital sodium (100 mg/kg). L3–L6 dorsal root ganglia was rapidly dissected (<10 min) in RNAse-free environment, and samples were immediately plunged into liquid nitrogen and then stored at –80°C until use.

### Small RNA sequencing and data analysis

Total RNA was extracted by TRIzol (Invitrogen, USA) from the L3–L6 dorsal root ganglion. The quality and quantity of RNA was measured by Nano Drop spectrophotometer (Thermo Scientific, USA) at the wavelength of 230, 260, and 280 nm. Small RNA library was constructed using mRNA-seq Library Prep Kit for Illumina (Vazyme Biotech, China) and subjected to deep sequencing using the Illumina HiSeq^TM^ 2500 platform. The quality of the sequencing data was evaluated using the Fast-QC tool (<http://http://www.bioinformatics.babraham.ac.uk/projects/fastqc/>), which provided the mass value distributions of the bases, the positional distributions of the mass values, and the GC content. The differentially expressed miRNAs between SNI rats and sham-operated rats were screened using EBSeq using the criteria Log2 fold change (FC) >1 or Log2FC <−1 and false discovery rate (FDR) <0.05. The target genes of differentially expressed miRNAs in the SNI rats were predicted using miRanda (<http://http://www.microrna.org/microrna/home.do>) (Score ≥150 and Energy <−20) and RNAhybrid (Energy <−25, <https://bibiserv.cebitec.uni-bielefeld.de/rnahybrid>). The target genes common between the lists output by these two tools were selected as the final target genes. Next, a target gene network map was generated using Cytoscape software (version 3.6.1, <https://cytoscape.org>). Functional annotation of these target genes was performed by Gene Ontology (GO) analysis with the threshold of FDR <0.05. In addition, Kyoto Encyclopedia of Genes and Genomes (KEGG) pathway enrichment analysis was performed using Fisher's exact test, and the overrepresented terms were screened at the cut-off value of FDR <0.05.

### Real-time PCR

Total RNA was isolated with TRIzol reagent (Invitrogen, USA). RNA quality and quantity were determined using a microspectrophotometer, and RNA integrity was evaluated by gel electrophoresis. The RNA was reverse-transcribed using a reaction mixture in accordance with the manufacturer's instructions (#K1622, Thermo, China). Five differentially expressed miRNAs screened by small RNA sequencing were validated by qPCR using SYBR green-based qPCR with ABI Q6 (Applied Biosystems Inc., USA), according to the manufacturer's instructions. RNA used for qPCR was also used for small RNA sequencing. U6 was used as the internal reference and the sequences of the primers are shown in Table 1. Relative expression levels were calculated using the 2^-ΔΔCt^ method (10).

**Table 1.** Primer sequence of real time PCR.

miRNAs	Primers
rno-miR-298-5p RT	5′ GTCGTATCCAGTGCGTGTCGTGGAGTCGGCAATTGCACTGGATACGACGGGAAGA 3′
rno-miR-298-5p F	5′ ATATATTGGCAGAGGAGGGCTGT 3′
rno-miR-298-5p R	5′ AGTGCGTGTCGTGGAGTCG 3′
rno-miR-1224 RT	5′ GTCGTATCCAGTGCGTGTCGTGGAGTCGGCAATTGCACTGGATACGACCTCCACC 3′
rno-miR-1224 F	5′ CTCTCATGTGAGGACTGGGGA 3′
rno-miR-1224 R	5′ AGTGCGTGTCGTGGAGTCG 3′
rno-miR-6215 RT	5′ GTCGTATCCAGTGCGTGTCGTGGAGTCGGCAATTGCACTGGATACGACCCTGGCT 3′
rno-miR-6215 F	5′ GCGGGTTTAGGGTTGCAGA 3′
rno-miR-6215 R	5′ AGTGCGTGTCGTGGAGTCG 3′
rno-miR-1249 RT	5′ GTCGTATCCAGTGCGTGTCGTGGAGTCGGCAATTGCACTGGATACGACTGAAGAA 3′
rno-miR-1249 F	5′ ATAATACGCCCTTCCCCCCCT 3′
rno-miR-1249 R	5′ AGTGCGTGTCGTGGAGTCG 3′
rno-miR-488-3p RT	5′ GTCGTATCCAGTGCGTGTCGTGGAGTCGGCAATTGCACTGGATACGACGACCAAG 3′
rno-miR-488-3p F	5′ GCGCAGTTGAAAGGCTGTTT 3′
rno-miR-488-3p R	5′ AGTGCGTGTCGTGGAGTCG 3′
U6-F	5′ CGATACAGAGAAGATTAGCATGGC 3′
U6-R	5′ AACGCTTCACGAATTTGCGT 3′′

### Intrathecal injection

A PE-10 polyethylene catheter (length, 15 cm) was intrathecally implanted into rats, and their hind limbs were paralyzed. Intrathecal drug administration was performed using a microinjection syringe attached to the intrathecal catheter. Five rats were included in each group. SNI rats were injected with miR-1224 agomir or agomir control (Ribobio, China). Agomir (20 μL, 5 nmol) was intrathecally injected each time and was administered on the day of operation and on days 4, 8, and 12 post-operation.

### Pain threshold determination

Allodynia was assessed by paw withdrawal threshold (PWT) using calibrated von Frey filaments (Stoelting, USA). In brief, a series of Von Frey filaments (force 2–26 g) were used to elicit the PWT. The plantar surface of the ipsilateral hind claw was stimulated for 6 s and it was positive if the rats showed foot contraction or foot licking, and the PWT was calculated as previous reported (11). Hyperalgesia was measured based on paw withdrawal latency (PWL) response to thermal stimulus using a thermal sensitive stimulator (Jixing Instrument Co., Ltd., China). The stimulator was set to 53°C. The rats were put into the observation box and timed with a stopwatch. PWL was recorded from the contact of hind paw with the hot plate to the end of licking the hind paw. Pain threshold was assessed 1 day before surgery and on days 1, 3, 5, 7, 14, and 21 post-operation. L3–L6 dorsal root ganglion was isolated at the end of behavioral testing, and rapidly frozen under −80°C.

### Statistical analysis

Data are reported as means±SE. SPSS software (Visual 22.0, IBM, USA) was used for statistical analysis. After verification of the normal distribution of data, the differences between the two groups were analyzed by Student's *t*-test. One-way ANOVA followed by Tukey's *post hoc* tests were performed to determine statistical differences in the pain threshold determination. P<0.05 was considered statistically significant.

## Results

### Differentially expressed miRNAs between SNI rats and sham-operated rats

**Figure 1.**
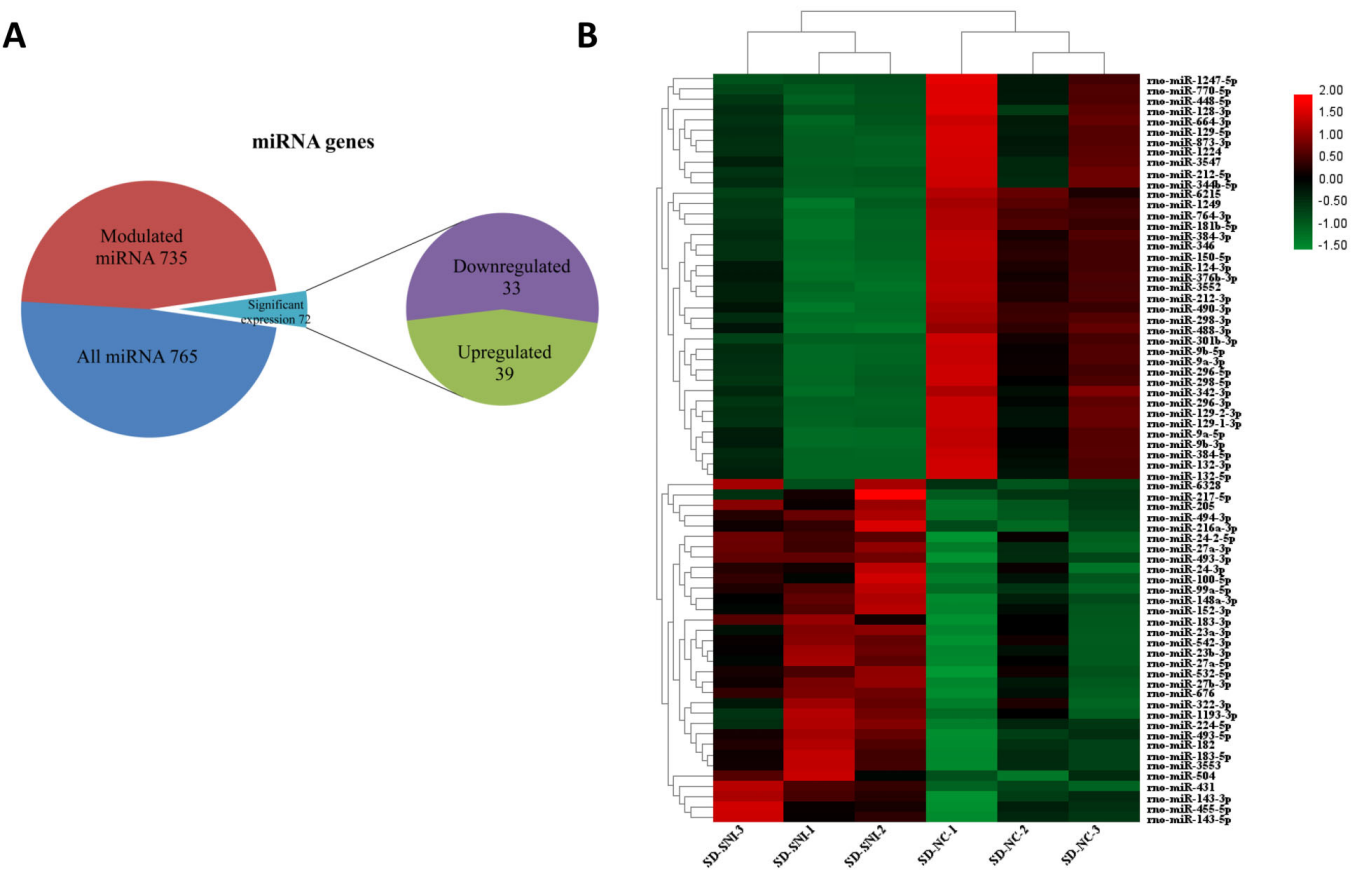
Differentially expressed microRNAs (miRNAs) between spared nerve injury (SNI) rats and sham-operated rats (NC). **A**, Expression of differentially expressed miRNAs; **B**, Hierarchical clustering of the differentially expressed miRNAs.

MiRNA sequencing results showed that the expression levels of 735 out of 765 miRNAs were modulated in SNI rats relative to those of sham-operated rats. Using the threshold values log2FC >1 or log2FC <−1, and FDR <0.05, we identified a total of 72 differentially expressed miRNAs comprising 33 upregulated and 39 downregulated miRNAs (Figure 1A). Hierarchical clustering analysis revealed differentially expressed miRNAs in the SNI and sham-operated groups (Figure 1B).

### Target gene analysis of differentially expressed miRNAs

**Figure 2.**
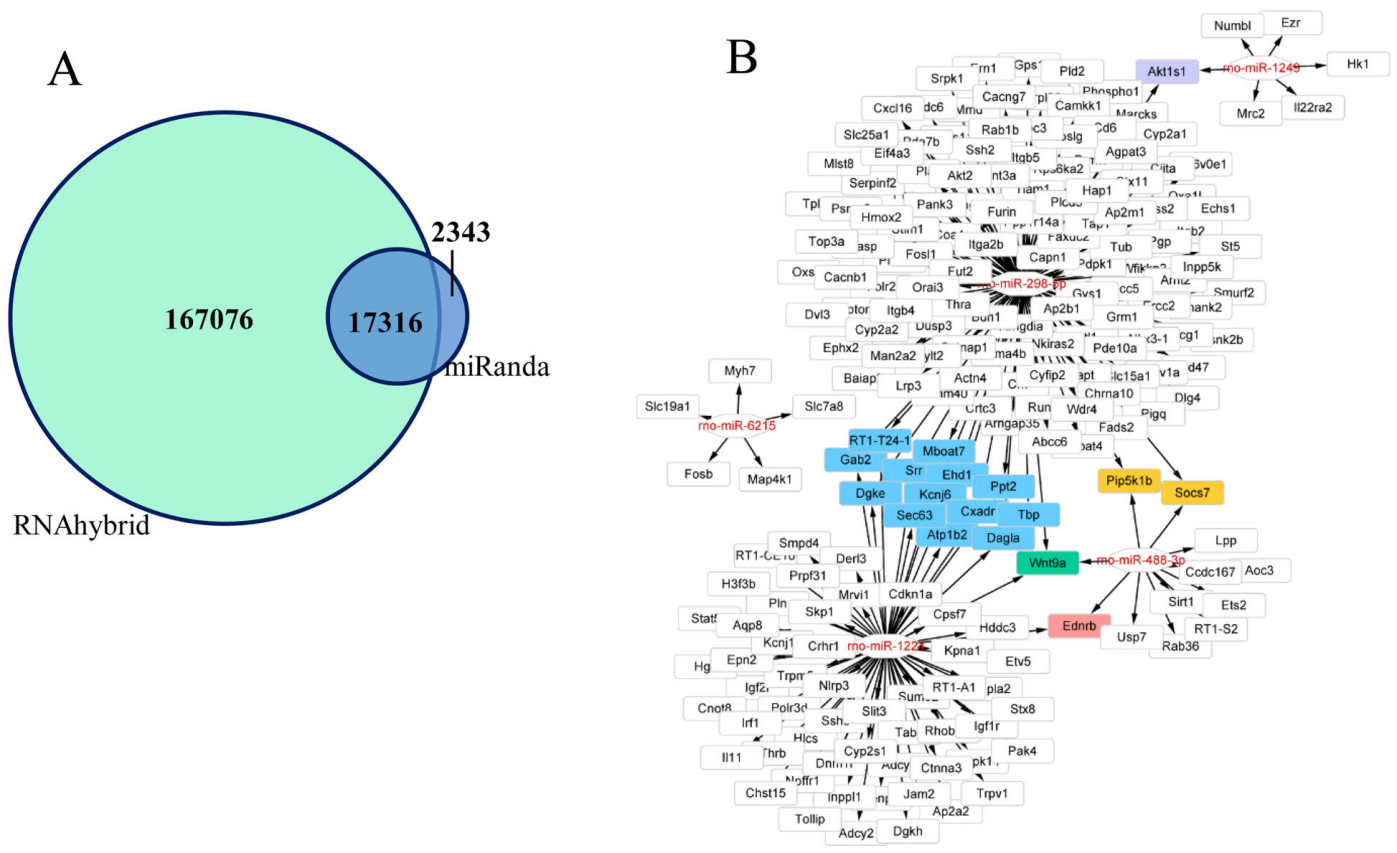
Target gene prediction of the differentially expressed microRNAs. **A**, Significantly differential expression of miRNA target gene prediction; **B**, Five most significantly differentially expressed miRNA target gene predictions; blue represents the common target gene of rno-miR-1224 and rno-miR-298-5p; purple represents the common target gene of rno-miR-1249 and rno-miR-298-5p; yellow represents the common target gene of rno-miR-488-3p and rno-miR-298-5p; red represents the common target gene of rno-miR-1224 and rno-miR-488-3p; green represents the common target gene of rno-miR-1224, rno-miR-488-3p, and rno-miR-298-5p.

The target genes of 72 differentially expressed miRNAs in the SNI rats were predicted using the tools miRanda and RNAhybrid. A total of 17,316 target genes were obtained (Figure 2A). Moreover, target gene analysis showed that rno-miR-298-5p, rno-miR-1224, and rno-miR-488-3p all targeted Wnt9a (Figure 2B). Wnt9a is a key gene in the Wnt pathway, which is known to be involved in neuropathic pain (12,13). miR-6215 targets Fosb, which promotes microglial activation and the production of pro-inflammatory cytokines, and thus controls neuropathic pain (14,15). In addition, we analyzed the target genes of miR-1249, which has been demonstrated to promote glioma cell proliferation by inhibiting Wnt/β-catenin signaling (16). The analysis identified a total of 207 potential target genes of the above-mentioned five miRNAs. These target genes have a wide range of functions, including cell proliferation, synaptic transmission, and phosphorylation.

### Functional annotation of target genes of differentially expressed miRNAs

**Figure 3.**
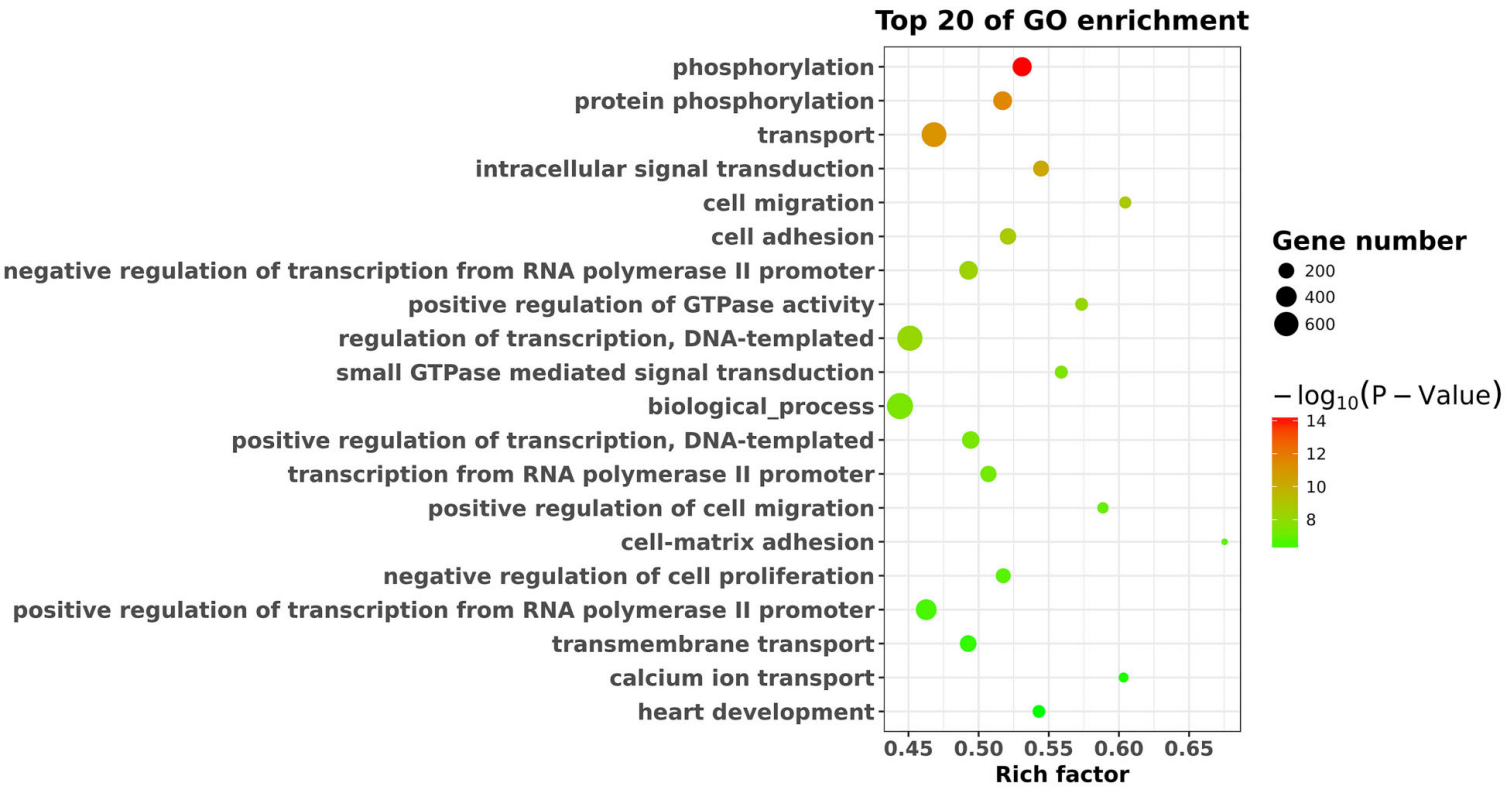
Functional enrichment analyses of the target genes of the differentially expressed miRNAs using Gene Ontology (GO) enrichment analysis. The top 20 biological processes are shown.

**Figure 4.**
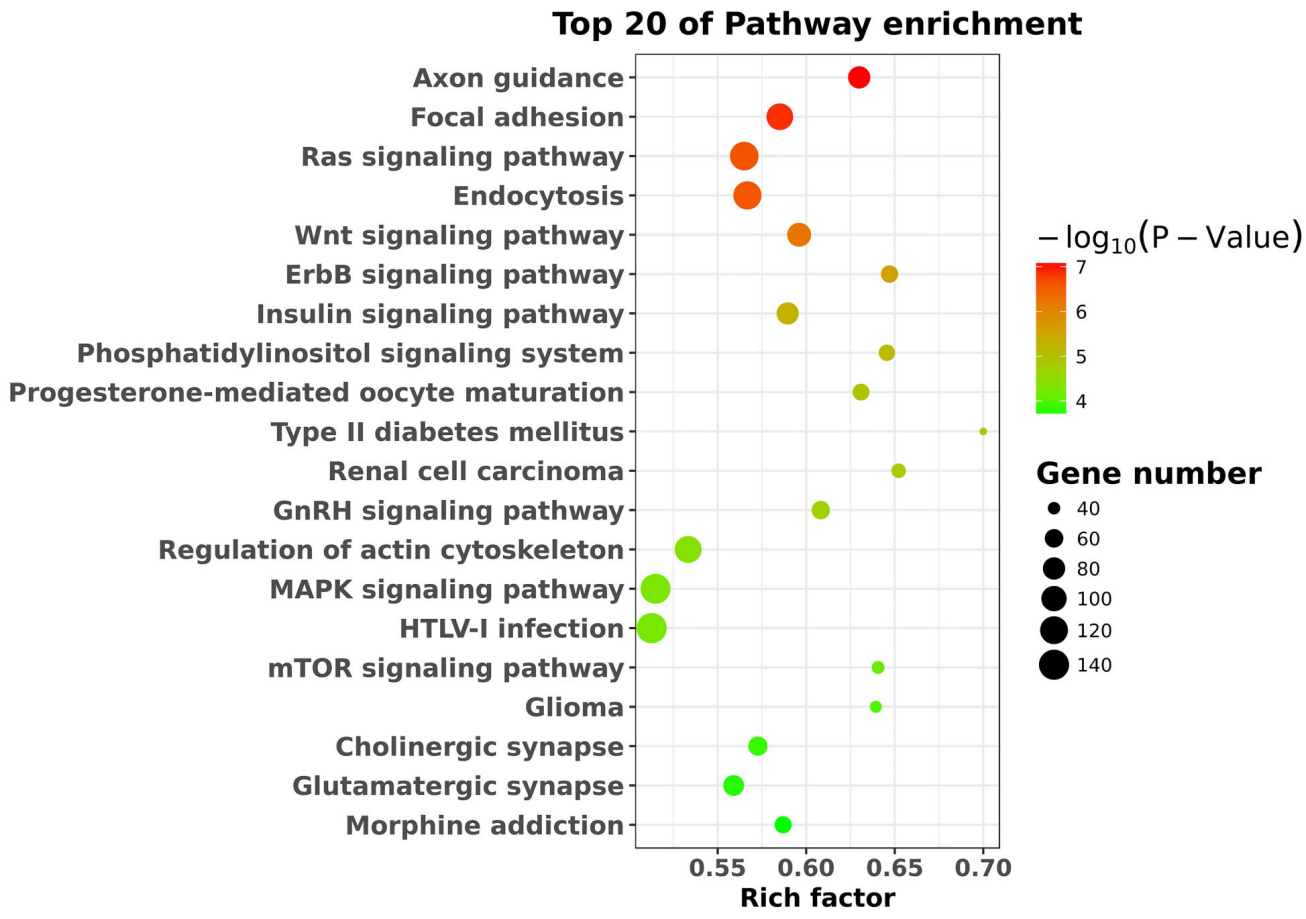
Pathway analysis of the target genes of differentially expressed miRNAs using Kyoto Encyclopedia of Genes and Genomes (KEGG) pathway enrichment analysis. The top 20 pathways are shown.

Functional annotation of target genes predicted by miRNAs revealed that they were significantly enriched in 56 GO-BP terms (P<0.05), including phosphorylation, transport, intracellular signal transduction, and cell migration (Figure 3). KEGG pathway enrichment analysis indicated that the target genes of the differentially expressed miRNAs were significantly enriched in terms of axon guidance, focal adhesion, and Ras and Wnt signaling pathways (Figure 4).

### qPCR validation of the candidate miRNAs

**Figure 5.**
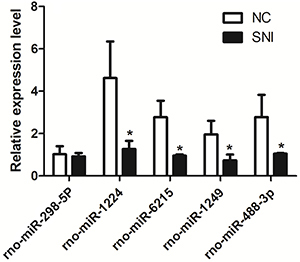
Real-time PCR validation of the differentially expressed miRNAs identified from small RNA sequencing. *P<0.05 (*t*-test), n=3. SNI: spared nerve injury rats; NC: sham-operated rats.

To validate the differentially expressed miRNAs, the best-suited candidate microRNAs (rno-miR-298-5p, rno-miR-1224, rno-miR-6215, rno-miR-1249, and rno-miR-488-3p) were analyzed by qPCR in three SNI and three sham-operated rats. The results of qPCR validation showed that rno-miR-298-5p expression was downregulated in SNI rats, although the results were not significantly different (P=0.684). Contrastingly, expression levels of rno-miR-6215 (P=0.015), rno-miR-1224 (P=0.030), rno-miR-1249 (P=0.038), and rno-miR-488-3p (P=0.048) were significantly downregulated in SNI rats compared to the control rats (Figure 5).

### Effect of differentially expressed miRNA on neuropathic pain

**Figure 6.**
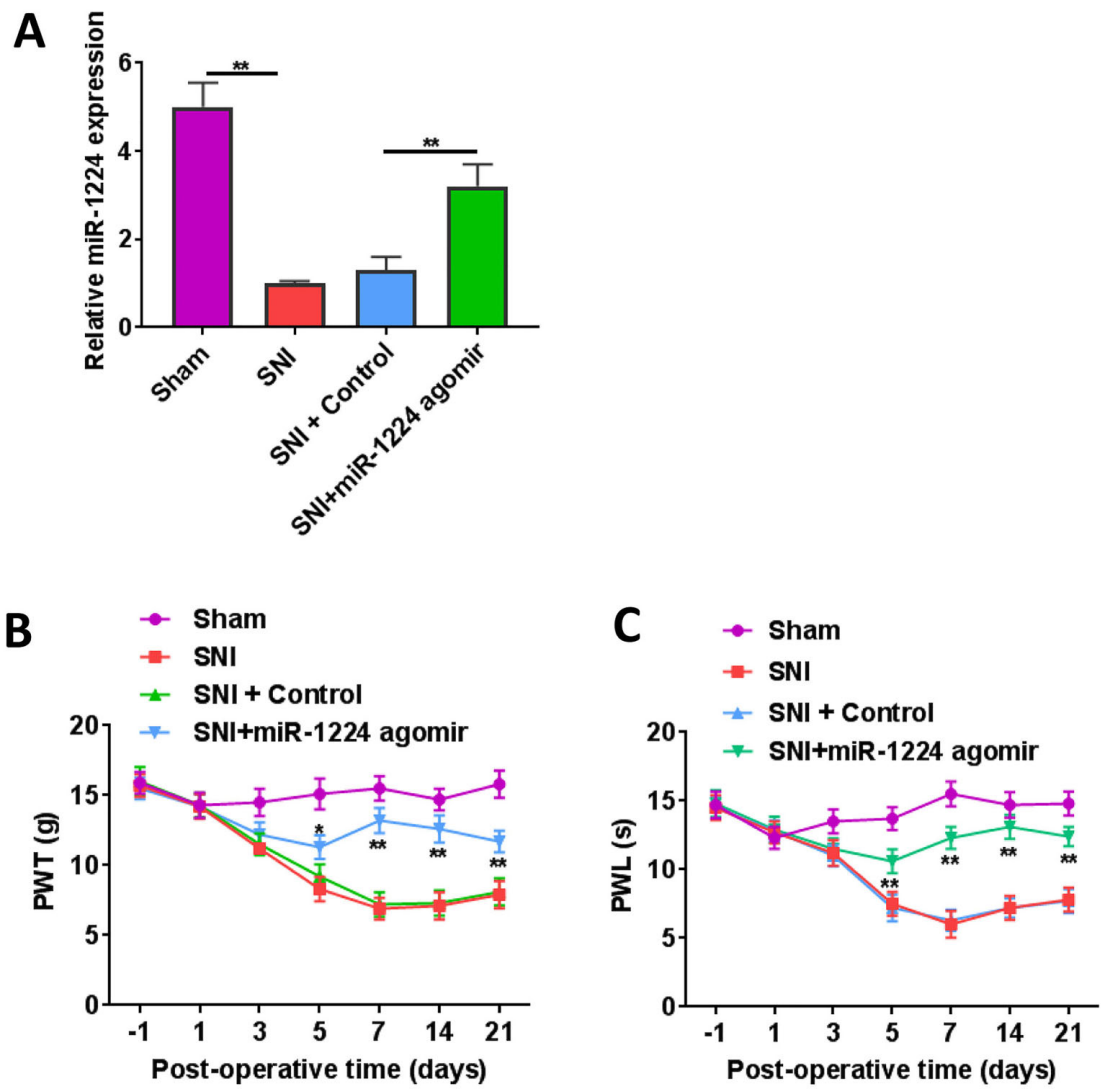
Effect of miR-1224 agomir on SNI-induced neuropathic pain. **A**, The expression of miR-1224 was verified in L3–L6 dorsal root ganglion using real time PCR on day 21 post-operation. **B**, The function of miR-1224 in mechanical allodynia was measured via paw withdrawal threshold (PWT). **C**, The function of miR-1224 in thermal hyperalgesia was measured via paw withdrawal latency (PWL), n=5 per group. *P<0.05, **P<0.01 miR-1224 agomir *vs* control (one-way ANOVA followed by *post hoc* Tukey’s test). SNI: spared nerve injury rats; NC: sham-operated rats.

To verify the roles of differentially expressed miRNAs in neuropathic pain, we conducted further analysis of the most downregulated miRNA, miR-1224. After the rats were intrathecally injected with miR-1224 agomir, miR-1224 was successfully overexpressed in SNI rats compared to those injected with the agomir control (Figure 6A). Moreover, miR-1224 agomir significantly alleviated SNI-induced neuropathic pain (Figure 6B and C).

## Discussion

Many patients suffer from neuropathic pain, and its pathogenesis, pathological diagnosis, and treatment remain difficult (17). Accumulating evidence shows that miRNAs play crucial roles in neuropathic pain. miRNAs have been reported to influence and regulate neuropathic pain (6–8). In the present study, we identified 72 differentially expressed miRNAs (33 upregulated miRNAs and 39 downregulated miRNAs) in SNI rats compared with sham rats. The results of qPCR validation showed that the expression levels of rno-miR-298-5p, rno-miR-6215, rno-miR-1224, rno-miR-1249, and rno-miR-488-3p were downregulated in SNI rats relative to sham rats. The above findings were consistent with the sequencing results. GO enrichment analysis suggested that the differentially expressed miRNAs could regulate genes involved in various processes, including phosphorylation, transport, intracellular signal transduction, and cell adhesion. KEGG pathway enrichment analysis indicated that the target genes of the differentially expressed miRNAs were significantly enriched in axon guidance, focal adhesion, and Ras and Wnt signaling pathways. The current findings could shed light on the mechanisms underlying the pathogenesis of neuropathic pain and help develop suitable treatment strategies.

A previous study showed that miRNA expression levels are altered in the blood of multiple rodent models of pain, including the SNI model of neuropathic pain (18). Zhou et al. (19) identified 12 miRNAs that were differently expressed in the L4-L5 spinal cord segments of SNI-induced rats 14 days after operation. In this study, we identified 72 differentially expressed miRNAs, including 33 upregulated and 39 downregulated miRNAs, in the L3–L6 dorsal root ganglia of SNI-induced rats 7 days after operation. Target gene prediction of the differently expressed miRNAs showed that rno-miR-298-5p, rno-miR-1224, and rno-miR-488-3p all targeted Wnt9a, a key gene in the Wnt signaling pathway (20–22). An increasing number of studies revealed that the Wnt signaling pathway, including the canonical Wnt/β-catenin signaling and non-canonical Wnt/Ryk signaling, contributes to neuropathic pain (13,23). During the nerve injury process, Wnt binds to its receptor and inhibits β-catenin phosphorylation by GSK-3, and the elevated levels of β-catenin associate with TCF4 to promote the induction of the Wnt target genes, which are responsible for neuropathic pain (24). Moreover, a number of drugs have been demonstrated to alleviate neuropathic pain via the Wnt pathway. For instance, cannabinoids could act as effective neuroprotective agents for neuropathic pain by inhibiting the Wnt/β-catenin signaling pathway (12). Additionally, a previous study revealed that miR-1249 promotes glioma cell proliferation by inhibiting Wnt/β-catenin signaling (16). The above-mentioned findings indicated that rno-miR-298-5p, rno-miR-1224, rno-miR-488-3p, and miR-1249 could act as mitigation targets for neuropathic pain by targeting and inhibiting the Wnt signaling pathway.

Moreover, our current results showed that downregulated miR-6215 can target FosB, which is also known as the G0/G1 switch regulatory protein 3. Previous studies showed FosB-/- mice inhibited microglial activation and the production of pro-inflammatory cytokines to control neuropathic pain (14,15). The levels of FosB in the dorsal hippocampus play an important role in both learning and hippocampal neurogenesis (25). Only persistent heterotopic pain in mice led to elevated FosB expression in the amygdala and the central nucleus of the spinal cord, and FosB levels remained elevated in the basal lateral amygdala of mice, which resulted in significant pain relief and persistent behavioral effects (15). The above findings suggested that miR-6215 is involved in SNI-induced neuropathic pain by targeting FosB.

Interestingly, miR-1224 levels were downregulated in the SNI rats compared to those in the sham rats, and treatment with miR-1224 agomir significantly alleviated neuropathic pain, as evidenced by its restoration of PWL and PWT, which were affected by SNI. Our findings were consistent with a study that found that miR-23a was downregulated in neuropathic pain rats compared to normal rats, and miR-23a agomir was reported to reduce neuropathic pain (26). However, several studies showed that the miRNAs that were downregulated in neuropathic pain groups compared to normal groups further aggravated neuropathic pain. For instance, miR-26a-5p expression was significantly downregulated in neuropathic pain established by a rat model of chronic sciatic nerve injury (CCI), however, miR-26a-5p overexpression exacerbated neuropathic pain (27). Furthermore, miRNA-146a-5p expression was significantly upregulated in the rat L4-L6 dorsal root ganglion and spinal dorsal horn after CCI surgery, while treatment with miRNA-146a-5p agomir attenuated CCI-induced neuropathic pain (7). These findings demonstrate the multiple functions and complex mechanisms of miRNAs during neuropathic pain.

The present study has certain limitations. The sample size was relatively small, and further studies should be conducted to elucidate the specific mechanisms underlying the effects of the candidate miRNAs identified. Additionally, it is impossible to measure miRNAs in patients' dorsal root ganglia. We also identified differentially expressed miRNAs and predicted their target genes but did not validate the target genes and the enriched metabolic pathways. However, the study of miRNA roles in the neuropathic pain rat models will enable further investigation in the pathogenesis of neuropathic pain and promote the development of novel therapeutics.

In conclusion, we reported changes in the expression of miRNAs in the dorsal root ganglion of SNI rats and suggested the potential roles of these alterations in the maintenance, development, and treatment of neuropathic pain.

## References

[1] Scholz J, Woolf CJ (2007). The neuropathic pain triad: neurons, immune cells and glia. Nat Neurosci.

[2] Descalzi G, Ikegami D, Ushijima T, Nestler EJ, Zachariou V, Narita M (2015). Epigenetic mechanisms of chronic pain. Trends Neurosci.

[3] Jensen TS, Baron R, Haanpaa M, Kalso Eija, Loeser JD, Rice ASC (2011). A new definition of neuropathic pain. Pain.

[4] Finnerup NB, Attal N, Haroutounian S, McNicol E, Baron R, Dworkin RH (2015). Pharmacotherapy for neuropathic pain in adults: a systematic review and meta-analysis. Lancet Neurol.

[5] Price TJ, Basbaum AI, Bresnahan J, Chambers JF, De Koninck Y, Edwards RR (2018). Transition to chronic pain: opportunities for novel therapeutics. Nat Rev Neurosci.

[6] Ni J, Gao Y, Gong S, Guo S, Hisamitsu T, Jiang X (2013). Regulation of mu-opioid type 1 receptors by microRNA134 in dorsal root ganglion neurons following peripheral inflammation. Eur J Pain.

[7] Wang Z, Liu F, Wei M, Qiu Y, Ma C, Shen L (2018). Chronic constriction injury-induced microRNA-146a-5p alleviates neuropathic pain through suppression of IRAK1/TRAF6 signaling pathway. J Neuroinflammation.

[8] Willemen HL, Huo XJ, Mao-Ying QL, Zijlstra J, Heijnen CJ, Kavelaars A (2012). MicroRNA-124 as a novel treatment for persistent hyperalgesia. J Neuroinflammation.

[9] Decosterd I, Woolf CJ (2000). Spared nerve injury: an animal model of persistent peripheral neuropathic pain. Pain.

[10] Livak KJ, Schmittgen TD (2001). Analysis of relative gene expression data using real-time quantitative PCR and the 2−ΔΔCT method. Methods.

[11] Chaplan SR, Bach FW, Pogrel JW, Chung JM, Yaksh TL (1994). Quantitative assessment of tactile allodynia in the rat paw. J Neurosci Methods.

[12] Nalli Y, Dar MS, Bano N, Rasool JU, Sarkar AR, Banday J (2019). Analyzing the role of cannabinoids as modulators of Wnt/beta-catenin signaling pathway for their use in the management of neuropathic pain. Bioorg Med Chem Lett.

[13] Liu S, Liu YP, Huang ZJ, Zhang YK, Song AA, Ma PC (2015). Wnt/Ryk signaling contributes to neuropathic pain by regulating sensory neuron excitability and spinal synaptic plasticity in rats. Pain.

[14] Fiore NT, Austin PJ (2018). Glial-cytokine-neuronal adaptations in the ventral hippocampus of rats with affective behavioral changes following peripheral nerve injury. Neuroscience.

[15] Bomholt SF, Mikkelsen JD, Blackburn-Munro G (2005). Normal hypothalamo-pituitary-adrenal axis function in a rat model of peripheral neuropathic pain. Brain Res.

[16] Fang B, Li G, Xu C, Hui Y, Li G (2018). MicroRNA miR-1249 downregulates adenomatous polyposis coli 2 expression and promotes glioma cells proliferation. Am J Transl Res.

[17] Baron R, Binder A, Wasner G (2010). Neuropathic pain: diagnosis, pathophysiological mechanisms, and treatment. Lancet Neurol.

[18] Qureshi RA, Tian Y, McDonald MK, Capasso KE, Douglas SR, Gao R (2016). Circulating microRNA signatures in rodent models of pain. Mol Neurobiol.

[19] Zhou J, Xiong Q, Chen H, Yang C, Fan Y (2017). Identification of the spinal expression profile of non-coding RNAs involved in neuropathic pain following spared nerve injury by sequence analysis. Front Mol Neurosci.

[20] Spater D, Hill TP, O'Sullivan R J, Gruber M, Conner DA, Hartmann C (2006). Wnt9a signaling is required for joint integrity and regulation of Ihh during chondrogenesis. Development.

[B21] Garriock RJ, Warkman AS, Meadows SM, D'Agostino S, Krieg PA (2007). Census of vertebrate Wnt genes: isolation and developmental expression of Xenopus Wnt2, Wnt3, Wnt9a, Wnt9b, Wnt10a, and Wnt16. Dev Dyn.

[22] Ali I, Medegan B, Braun DP (2016). Wnt9A induction linked to suppression of human colorectal cancer cell proliferation. Int J Mol Sci.

[23] Moreau N, Mauborgne A, Couraud PO, Romero IA, Weksler BB, Villanueva L (2017). Could an endoneurial endothelial crosstalk between Wnt/β-catenin and Sonic Hedgehog pathways underlie the early disruption of the infra-orbital blood-nerve barrier following chronic constriction injury?. Mol Pain.

[24] Fernandes V, Sharma D, Vaidya S, P A S, Guan Y, Kalia K (2018). Cellular and molecular mechanisms driving neuropathic pain: recent advancements and challenges. Expert Opin Ther Targets.

[25] Manning CE, Eagle AL, Kwiatkowski CC, Achargui R, Woodworth H, Potter E (2019). Hippocampal subgranular zone FosB expression is critical for neurogenesis and learning. Neuroscience.

[26] Pan Z, Shan Q, Gu P, Wang XM, Tai LW, Sun M (2018). miRNA-23a/CXCR4 regulates neuropathic pain via directly targeting TXNIP/NLRP3 inflammasome axis. J Neuroinflammation.

[27] Zhang Y, Su Z, Liu HL, Li L, Wei M, Ge DJ (2018). Effects of miR-26a-5p on neuropathic pain development by targeting MAPK6 in in CCI rat models. Biomed Pharmacother.

